# Measles Vaccination Coverage and Anti-Measles Herd Immunity Levels in the World and WHO Regions Worsened from 2019 to 2023

**DOI:** 10.3390/vaccines13020157

**Published:** 2025-02-05

**Authors:** Pedro Plans-Rubió

**Affiliations:** College of Physicians of Barcelona, 08017 Barcelona, Spain; pedro.plans@yahoo.es

**Keywords:** measles vaccination, measles elimination and eradication, WHO regions, two-dose measles coverage, MCV1, MCV2, measles zero-dose coverage, anti-measles herd immunity, measles prevention strategies

## Abstract

Objectives: The objectives of this study were as follows: to determine mean percentages of measles vaccination coverage with zero, one and two doses of vaccine and anti-measles herd immunity levels in World Health Organization (WHO) regions in 2023; to assess variations in measles vaccination coverage and anti-measles herd immunity-related indicators from 2019 to 2023; and to assess whether zero-dose measles vaccination coverage indicators were on track to achieve the Immunization Agenda 2030 objective. Methods: Mean percentages of vaccination coverage with two, one and zero doses of measles vaccine in WHO regions in 2023 were calculated using data from the WHO/UNICEF global and regional immunization information system. Results: In 2023, the global mean two-dose measles vaccination coverage was 65.3%, and mean two-dose vaccination coverage was lower than 95% in all WHO regions; the mean prevalence of measles-protected individuals in the target vaccination population was 87.6%, and anti-measles herd immunity levels in the target vaccination population were sufficient to block the transmission of measles viruses with greater transmissibility (R_o_ ≥ 15) only in the Western Pacific and European WHO regions. The global mean two-dose measles vaccination coverage decreased by 3.7% from 2019 to 2023. In 2023, the mean zero-dose measles coverage and number of zero-dose measles children were, respectively, 36.7% and 40.6% greater than the values required to be on track to achieve the 2030 objective. Conclusion: This study found that all measles-vaccination-coverage-related indicators worsened from 2019 to 2023, and the zero-dose measles vaccination coverage and number of zero-dose measles children in 2023 were not on track to achieve the AI2030 objective. Interventions to increase routine two-dose measles vaccination coverage should be developed in all WHO regions.

## 1. Introduction

In 2020, the 73rd World Health Assembly endorsed the Immunization Agenda 2030 (IA2030), envisioning “a world where everyone, everywhere, at every age, fully benefits from vaccines for good health and well-being” [1]. In 2011, the Global Vaccine Action Plan was proposed to eliminate measles in at least five WHO regions by 2020 [2]. The IA2030 agenda has committed to eliminating measles in at least five of the six WHO regions by 2030 [1,2].

Measles can be eliminated in all WHO regions and eradicated in the world because measles has only human reservoir, effective measles vaccines are available, and intensive epidemiological surveillance using highly sensitive and specific diagnostic tests can detect all new measles cases [3]. In fact, measles was eliminated in the Americas region in 2016 [4]. Unfortunately, measles re-emerged in Brazil and Venezuela in 2018, the Americas region lost its elimination status in 2019, and measles cases and outbreaks occurred in all WHO regions during the 2019−2023 period [4,5,6,7,8]. In the European region, a total of 9010 measles cases, 4259 hospitalizations and 2 deaths due to measles were reported by European countries to the WHO’s centralized information system for infectious diseases (CISID) in 2023 [6,7,8]. Since 2019, measles elimination has not been verified in any WHO region [2,5].

The WHO considers that achieving and maintaining percentages of routine vaccination coverage of at least 95% with two doses of the measles-containing vaccine (MCV) is the key intervention to achieve measles elimination and eradication [9,10,11,12]. Routine measles vaccination includes two doses of measles–mumps–rubella (MMR) vaccine, which must be administered to all children aged 12–15 months and 3–15 years [9,12,13,14,15]. High percentages of routine measles vaccination can generate both individual measles protection and sufficient population herd immunity to block measles transmission in the community [9,10,11,12,16].

A study carried out in 2019 [10] found that only 14.4% of countries worldwide had a two-dose measles vaccination coverage ≥ 95%, and anti-measles herd immunity levels in the target vaccination population were not sufficient against measles viruses with basic reproduction numbers (R_o_) ≥ 10 in the African and Eastern Mediterranean regions, measles viruses with R_o_ ≥ 11 in the Western Pacific region and measles viruses with R_o_ ≥ 13 in the Americas, European and South-East Asia regions [10].

The objectives of this study were as follows: (1) to determine the vaccination coverage for zero, one and two doses of measles vaccine and anti-measles herd immunity levels in countries and WHO regions in 2023; (2) to assess variations in measles vaccination coverage and anti-measles herd immunity-related indicators in WHO regions from 2019 to 2023; and (3) to assess whether zero-dose and measles vaccination coverage indicators were on track to achieve the Immunization Agenda 2030 objective.

## 2. Methods

### 2.1. Mean Vaccination Coverage with Zero, One and Two Doses of Measles Vaccine in the WHO Regions in 2023

The mean percentages of vaccination coverage for the first and second dose of measles-containing vaccine (MCV1, MCV2) were determined for different regions of the WHO using the information from the WHO/UNICEF global and regional immunization information system [17]. WHO considers six regions: African region, Americas region, Eastern Mediterranean region, European region, South-East Asia region and Western Pacific region (WPR) [2,17]. WHO and UNICEF estimate coverage with the first and second measles-containing vaccine (MCV1 and MCV2) doses delivered by routine immunization services for all countries using annual administrative estimates and vaccination coverage surveys [18]. Percentages of vaccination coverage for the MCV1 vaccine in different countries were estimated among children aged 1 year or among children aged 2 years, when the MCV1 was given to children aged ≥ 1 year [18]. Percentages of vaccination coverage for the MCV2 vaccine in different countries were estimated among children at the recommended age, according to national immunization schedules [18].

### 2.2. Anti-Measles Herd Immunity Levels in the Target Vaccination Population in the WHO Regions in 2023

Anti-measles herd immunity levels in the target vaccination populations were determined in different WHO regions in 2023 from the country-based mean vaccination coverage with one and two doses of measles vaccine, and the effectiveness for one and two doses of measles vaccine [10]. In this study, values of effectiveness in preventing measles cases of 92% and 95% were assumed for one and two doses of the measles vaccine [19].

Herd immunity against measles viruses with R_o_ values of 10, 11, 12, 13, 14, 15, 16, 17, 18, 19 and 20 was considered established in the world and in different WHO regions when the mean prevalence of individuals with vaccine-induced measles protection was higher than 90%, 90.9%, 91.7%, 92.3%, 92.9%, 93.3%, 93.8%, 94.1%, 94.4%, 94.7% and 95%, respectively [10,11].

The percentage of countries in different WHO regions with sufficient herd immunity against measles viruses with R_o_ values equal to or lower than 10, 12, 15, 18, 19 and 20 were assessed by counting the number of countries in each region with a prevalence of individuals with vaccine-induced measles protection in the target vaccination population higher than 90%, 91.7%, 93.3%, 94.4%, 94.7% and 95%, respectively [10,11].

### 2.3. Assessment of Whether Zero-Dose Measles Vaccination Indicators in 2023 Were on Track to Achieve the Immunization Agenda 2030 Objective

In this study, tracks required from 2019 to 2030 to achieve a 50% reduction by 2030 were determined for three zero-dose measles coverage indicators: (1) estimated number of children who did not receive the first dose of measles-containing vaccine (MCV1) [20]; (2) mean MCV1-based zero dose coverage; and (3) mean zero dose coverage determined from one and two doses of measles vaccine coverage [10].

Immunization Agenda 2030 proposes a 50% reduction in the number of zero-dose children observed in 2019 by 2030 [20]. For operational purposes, WHO/UNICEF defines zero-dose children for measles vaccination as those who lack the MCV1 vaccine [20]. The track required from 2019 to 2030 to achieve the AI2030 objective of number of zero-dose children was determined by considering the WHO/UNICEF estimated number of children who did not receive the MCV1 vaccine in 2019 (22.2 million) [20], and a 50% lower number (9.65 million) in 2030. To assess whether the number of zero-dose children was on track to achieve the 2030 goal, the estimated number of zero-dose children in 2023 was compared with the number required to be on track.

The track required from 2019 to 2030 for mean MCV1-based zero-dose measles coverage to achieve a 50% reduction by 2030 was determined by considering the mean MCV1-based zero-dose coverage (12.4%) observed in 2019 [10] and a 50% lower coverage (6.2%) in 2030. This analysis can be considered similar to the analysis based on the number of zero-dose measles children because WHO/UNICEF estimates the number of zero-dose children from 100−MCV1 coverage [4,20]. To assess whether the zero-dose measles coverage in 2023 was on track to achieve the 2030 goal, the coverage in 2023 was compared with the coverage required to be on track.

The track required to achieve a 50% reduction from 2019 to 2030 for zero-dose measles coverage (from two-dose and one-dose coverage) was determined by considering the mean zero-dose coverage obtained in 2019 (12.4%) [10] and a 50% lower level in 2030 (6.2%). To assess whether the mean zero-dose measles coverage in 2023 was on track to achieve the 2030 goal, the coverage in 2023 was compared with the mean two-dose coverage required to be on track.

### 2.4. Statistical Analysis

Microsoft Excel (Microsoft Corporation, Redmond, WA, USA) was used to calculate the following: (1) the mean percentage of vaccination coverage with the MCV1 and MCV2 vaccines in 2023 in different WHO regions; (2) the percentages of vaccination coverage with zero, one and two doses of measles vaccine in different countries of the world; (3) the mean percentages of vaccination coverage with zero, one and two doses of measles vaccine in regions of the WHO; (4) the prevalence of vaccine-induced measles protection in individuals in the target measles vaccination population in different countries of the world on 2023; and (5) the mean prevalence of vaccine-induced measles-protected individuals in the target measles vaccination population in regions of the WHO. Microsoft Excel (Microsoft Corporation, Redmond, WA, USA) was used to assess the establishment of herd immunity against measles viruses in countries of the world and regions of the WHO in 2023.

## 3. Results

### 3.1. Mean Percentages of Routine Measles Vaccination Coverage in Countries of the World and Regions of the WHO in 2023

In 2023, the worldwide mean vaccination coverage with the MCV1 and MCV2 measles vaccines were 85.2 and 77.1%, respectively (Table 1). The mean MCV1 vaccination coverage was lower than 95% in all WHO regions, except the Western Pacific region (97%) (Table 1). The mean MCV2 vaccination coverage was lower than 95% in all WHO regions, except the Western Pacific region (95.5%) (Table 1).

### 3.2. Mean Percentages of Vaccination Coverage with Zero, One and Two Doses of Measles Vaccines in the World and Regions of the WHO in 2023

The worldwide mean percentages of vaccination coverage for zero, one and two doses of measles vaccine were 65.3%, 27.8% and 6.9%, respectively (Table 2). The global mean two-dose measles vaccination coverage found in this study (65.3%) was 31% lower than the 95% objective proposed by the WHO.

The highest mean two-dose measles vaccination coverage was found in the Western Pacific region (93.2%) and the lowest one in the African region (48.1%) (Table 2). The highest mean one-dose measles vaccination coverage was found in the South-East Asia region (46.5%) and the lowest one in the Western Pacific region (6.7%) (Table 2). The highest mean zero-dose measles vaccination coverage was found in the African region (21.1%), while mean percentages of zero-dose coverage lower than 1% were found in the Western Pacific, South-East Asia and European regions (Table 2).

The two-dose measles vaccination coverage was ≥95% in only 17 (8.7%) countries of the world, and it was ≥ 90% in 41 (21%) countries (Table 2).

In the African region, 0% of the countries had a two-dose measles vaccination coverage higher than or equal to 90%, while in the Eastern Mediterranean region, 22.7% and 40.9% of the countries had a two-dose measles vaccination coverage higher than or equal to 95% and 90%, respectively (Table 2).

### 3.3. Anti-Measles Herd Immunity Levels in Countries of the World and Regions of the WHO in 2023

The worldwide mean prevalence of vaccine-induced measles protection found in this study was 87.6% (Table 2). Worldwide measles protection levels were not sufficient to block the transmission of measles viruses with R_o_ values equal to or greater than 10, as herd immunity against these viruses can be established with a 90% prevalence (Table 2).

The mean (per country) prevalence of measles protection ranged from 74% in African region to 94.1 in the European region and 94.6% in the Western Pacific region (Table 2). Based on the regional mean prevalence of vaccine-induced measles protection, anti-measles herd immunity levels (target vaccination population) were sufficient to block the transmission of measles viruses with greater transmissibility (R_o_ ≥ 15) only in the Western Pacific and European WHO regions (Table 2). Anti-measles herd immunity was established against measles viruses with R_o_ ≤ 18 (94.4% required) in the Western Pacific region; against viruses with R_o_ ≤ 16 (93.8% required) in the European region; against viruses with R_o_ ≤ 14 (92.9% required) in the South-East Asia region; against viruses with R_o_ ≤ 12 (91.7% required) in the Americas region; and against viruses with R_o_ ≤ 11 (90.9% required in the Eastern Mediterranean region (Table 2). Anti-measles herd immunity was not established against measles viruses with R_o_ ≥ 19 in any WHO region because the mean prevalence of vaccine-induced immune protection was lower than 94.7% in all regions (Table 2). In the African region, anti-measles herd immunity was not established against viruses with R_o_ ≥ 10 because its mean prevalence of vaccine-induced immune protection as lower than 90% (Table 2).

The percentage of countries with sufficient herd immunity against measles viruses with R_o_ values equal to or lower than 15 was 41.5%, while 23.1% of the countries had sufficient herd immunity against measles viruses with R_o_ values equal to or lower than 18 (Table 2).

The Eastern Mediterranean, Europe, South-East Asia and Western Pacific regions had the best country-based anti-measles herd immunity profiles, while the African region had the worst profile (Table 2).

### 3.4. Variation for Routine Measles Vaccination-Related Indicators in Countries of the World and Regions of the WHO from 2019 to 2023

The study found that five measles vaccination coverage-related indicators worsened and one indicator improved from 2019 to 2023 (Table 3).

The mean global two-dose measles vaccination coverage decreased by 3.7% and the mean global zero-dose measles vaccination coverage increased by 7.8% from 2019 to 2023 (Table 3). The mean global one-dose vaccination coverage improved by 7.8% from 2019 to 2023 (Table 3).

The percentage of countries with two-dose measles vaccination coverage ≥95% and ≥ 90% decreased by 39.6% and 18.6%, respectively, from 2019 to 2023 (Table 3). The percentage of countries where all children had received at least one dose of measles vaccine (without zero-dose children) decreased by 24.8% from 2019 to 2023 (Table 3).

The global mean two-dose measles vaccination coverage decreased from 2019 to 2023 in the South-East Asia, Eastern Mediterranean and Americas regions, while it improved in the other regions (Table 3). The variation for this indicator ranged from −35% in the South-East Asia region to 30.9% in the Western Pacific region (Table 3).

The mean zero-dose measles vaccination coverage decreased from 2019 to 2023 in all WHO regions except in the African region (Table 3). The variation for this indicator ranged from −96.5% in the Western Pacific region to 24.9% in the African region (Table 3).

The percentage of countries with two-dose measles vaccination coverage ≥ 95% decreased between 2019 and 2023 in all WHO regions (Table 3). The variation for this indicator ranged from −4.6% in the Eastern Mediterranean region to −100% in the African region (Table 3).

The percentage of countries with two-dose measles vaccination coverage ≥ 90% decreased between 2019 and 2023 in all WHO regions, except in the Eastern Mediterranean and South-East Asia regions (Table 3). The variation for this indicator between 2019 and 2023 ranged from 7.3% in the Eastern Mediterranean region to −100% in the African region (Table 3).

The percentage of countries without zero-dose measles vaccination children because all children had received one or two doses of measles vaccine decreased from 2019 to 2023 in all WHO regions, except in the European and Western Pacific regions (Table 3). The variation for this indicator between 2019 and 2023 ranged from 0% in the European and Western Pacific regions to −100% in the African region (Table 3).

### 3.5. Variation for Anti-Measles Herd Immunity-Related Indicators in Countries of the World and Regions of the WHO from 2019 to 2023

The study found that all anti-measles herd immunity-related indicators assessed in this study worsened in the world from 2019 and 2023 (Table 3). The mean prevalence of individuals with vaccine-induced measles immunity decreased by 0.6%, and the percentage of countries with herd immunity against measles viruses with R_o_ values ≤ 19 decreased by 100% from 2019 to 2023 (Table 3).

The mean prevalence of individuals in the target vaccination population with vaccine-induced measles immunity increased in the Western Pacific, Eastern Mediterranean, European and Americas regions, while it decreased in the Africa and South-East Asia regions between 2019 and 2023 (Table 3). The variation for this indicator between 2019 and 2023 ranged from 6.5% in the Western Mediterranean region to −4.6% in the African region (Table 3).

### 3.6. Assessment of Whether Zero-Dose Measles Vaccination Indicators in 2023 Were on Track to Achieve the IA2030 Objective Reduction by 2030

**Figure 1:**
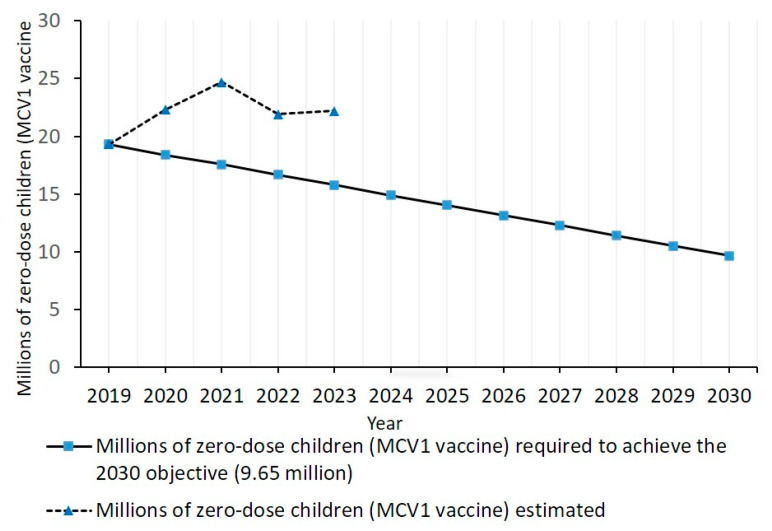
Number of zero-dose measles children (did not receive the MCV1 vaccine) estimated from 2019 and 2023, and number of zero-dose children required from 2019 to 2030 to achieve the IA 2030 objective (9.65 million).

**Figure 2:**
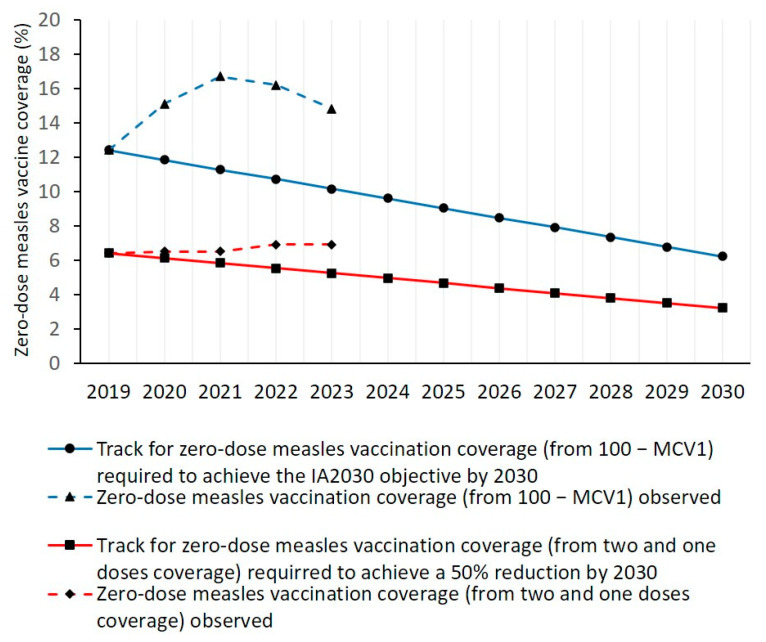
Track to achieve a 50% reduction from 2019 to 2030 for mean MCV1-based zero-dose measles vaccination coverage, and MCV1-based zero-dose coverage observed from 2019 to 2023. Track to achieve a 50% reduction from 2019 to 2030 for mean zero-dose measles vaccination coverage determined from two and one doses of measles vaccination, and zero-dose coverage observed in this study for 2023.

In 2023, the three indicators of zero-dose measles coverage assessed in this study were not on track to achieve a 50% reduction in their 2019 values by 2030. The number of zero-dose measles children estimated by the WHO/UNICEF, the mean MCV1-based zero-dose coverage and the two-dose measles coverage were 40.6%, 46,5% and 36.7% greater than the values required to be on track to achieve the 2030 objective (Figure 1 and Figure 2).

The number of zero-dose measles children estimated by WHO/UNICEF in 2023 (22.2 million) [20] was not on track to achieve the 2030 objective of 9.65 million (Figure 1). To be on track, the number of zero-dose measles children should be reduced by 877,273 per year from 2019 to 2030 (Figure 1). The number of zero-dose measles children estimated in 2023 was 40.6% greater than the number required (15.8 million) to be on track. The number of zero-dose measles children estimated by WHO/UNICEF for 2020 (22.3 million), 2021 (24.7 million) and 2022 (21.9 million) [4,20,21] were also out of track to achieve the IA2030 objective (Figure 1).

In 2023, the mean zero-dose measles vaccination coverage determined from the mean MCV1 coverage (14.8%) was not on track to achieve the 2030 objective of 6.2% (Figure 2). To be on track, the MCV1-based zero-dose coverage should be reduced by 0.56% per year from 2019 to 2030. In 2023, the mean MCV1-based zero-dose coverage was 46.5% greater than the coverage required (10.1%) to be on track to achieve the 2030 objective (Figure 2). The mean MCV1-based zero-dose coverage in 2020, 2021 and 2022 were also off track (Figure 2).

In 2023, the mean zero-dose measles vaccination coverage determined from the coverage for two and one doses of measles vaccine (6.9%) was not on track to achieve the 2030 objective of 3.2% (Figure 2). To be on track, the zero-dose coverage should be reduced by 0.29% per year from 2019 to 2030. In 2023, the mean zero-dose coverage was 36.7% greater than the coverage required (5.2%) to be on track to achieve 2030 (Figure 2). The mean zero-dose coverages in 2020, 2021 and 2022 were also off track (Figure 2).

## 4. Discussion

This study found four key results for 2023: (1) The global means for two-dose, one-dose and zero-dose measles vaccination coverage were 65.3%, 27.8% and 6.9%, respectively. (2) Mean percentages of two-dose measles vaccination coverage were lower than ≥95% in all WHO regions. (3) The mean prevalence of measles-protected individuals in the target vaccination population was 87.6%. (4) Anti-measles herd immunity levels in the target vaccination population was sufficient to block the transmission of measles viruses with greater transmissibility (R_o_ ≥ 15) only in the Western Pacific and European WHO regions.

In addition, this study found that measles vaccination coverage and anti-measles herd immunity-related indicators worsened from 2019 to 2023. The mean measles vaccination coverage increased by 7.8%. The percentage of countries with two-dose measles vaccination coverage ≥ 95% decreased by 39.6%. The mean prevalence of measles-protected individuals in the target vaccination population decreased by 0.6%. The mean MCV1 coverage and the number of zero-dose measles vaccine children in 2023 were not on track to achieve the AI2030 objective of zero-dose measles vaccination coverage.

The global mean zero-dose measles vaccination coverage found in this study in 2023 was 6.9%, and it increased by 7.8% from 2019 to 2023. The worldwide mean zero-dose measles coverage found in 2023 can be explained by the high mean zero-dose coverage in countries in the African region (21.1%), which contrasted with the <3% mean zero-dose coverage in the other WHO regions. The 7.8% increase in this indicator between 2019 and 2023 can be explained by the 24.9% increase in the African region. In fact, the mean zero-dose measles coverage improved in all WHO regions from 2019 to 2023, except in the African region.

In addition, the study found that the percentage of countries without zero-dose measles children because all children had received at least one dose of measles vaccine was only 9.7%. However, the variation for this indicator from 0% in the African region to 22% in the Eastern Mediterranean region shows that reducing the zero-dose measles vaccine children must be a priority in all countries and WHO regions.

Measles meets the criteria for disease eradication, but the results found in this study for 2023 showed that routine measles vaccination programs had not recovered from COVID-19 pandemic disruptions, and progress toward measles elimination in most countries and in all WHO regions had slowed. A similar result was found by WHO/UNICEF [20], and inadequate progress toward measles eradication was found in prior evaluations carried out in 2016 [22], 2019 [10], 2021 [21] and 2022 [4]. The midterm evaluation of the Global Measles Strategic Plan 2012–2020 carried out in 2016 [22] considered that progress toward measles eradication was inadequate in 2016 due to a lack of political will as well as country ownership, reflected in insufficient resources.

The IA2030 agenda has committed to eliminating measles in at least five of the six WHO regions by 2030, and one of the key IA2030 objectives is to reduce the number of children who have not received at least one measles vaccine (zero-dose measles children) by 50% from 2019 to 2030 [1,20]. However, this study found that the three indicators of zero-dose measles coverage assessed were not on track for achieving a 50% reduction from 2019 to 2030. In 2023, the number of zero-dose measles children estimated by the WHO/UNICEF and the mean MCV1-based zero-dose coverage and the two-dose measles coverage were 40.6%, 46.5% and 36.7% greater than their values required to be on track to achieve the 2030 objective.

The priority prevention strategy to eliminate measles in different WHO regions is to achieve and maintain percentages of two-dose measles vaccination coverage equal to or greater than 95% for three reasons. Firstly, achieving and maintaining a routine measles vaccination coverage ≥ 95% can generate sufficient population immunity to establish anti-measles herd immunity in the community [10,11,23]. Secondly, it is a critical strategy in the path toward measles elimination because it can be achieved only in countries with strong measles vaccination programs and strong primary healthcare services [20,24]. Thirdly, the strategy to increase measles vaccination coverage with SIAs has limitations that make it insufficient for achieving and maintaining measles elimination [10,11,25].

In each country and WHO region, a routine measles vaccination coverage with two doses of measles vaccine coverage of at least 95% should be achieved in every birth cohort and in different areas and communities to ensure sufficient population immunity to establish anti-measles herd immunity against measles viruses [1,2,3,10,11,25]. Based on the results of this study, three measles prevention strategies should be developed depending on the two-dose routine measles vaccination coverage and anti-measles vaccine-induced herd immunity levels achieved in 2023. In the European and Western Pacific regions, the main objective should be to increase their mean two-dose measles vaccination coverage from 86–93% to ≥95% to avoid cases and outbreaks generated by viruses imported from endemic countries.

In the Eastern Mediterranean, Americas and South-East Asia regions, the main objective should be to increase their 53–72% mean two-dose measles vaccination coverage to ≥95% and to increase anti-measles herd immunity levels to block transmission of measles viruses with greater transmissibility.

In the African region, great efforts and international support are necessary to increase the two-dose measles vaccination coverage in the African region from 48% to ≥95%, reduce the number of zero-dose measles vaccine children and increase anti-measles herd immunity levels. This study found that insufficient global mean levels for measles vaccination-related and anti-measles herd immunity-related indicators depended greatly on the situation in the African region. Therefore, the global measles eradication objective depends greatly on improving the measles situation in the African region.

The following interventions can be used to increase routine two-dose measles vaccination coverage to ≥95% and to increase anti-measles herd immunity levels in all countries and WHO regions: (1) implement advanced vaccination programs [26,27]; (2) increase routine two-dose measles vaccination coverage from 95% to 97% [11]; (3) develop measles screening and vaccination programs to reach susceptible individuals and populations with low measles immunity levels [10,11,25,28,29]; (4) develop supplementary vaccination activities [20,30,31]; (5) implement interventions to increase measles vaccination access and vaccine provision [32]; (6) implement interventions to reduce measles vaccination hesitancy [33,34,35,36]; (7) implement interventions to increase healthcare provider engagement [37]; and (8) implement compulsory measles vaccination [38,39].

Screening and vaccination programs can be developed to reach susceptible individuals and populations with low levels of measles protection. This strategy can achieve and maintain herd immunity levels against measles viruses with greater transmissibility by (i) immunizing all susceptible individuals in the population or (2) immunizing susceptible individuals in areas and population groups with low measles protection levels [10,11,25,28,40]. However, seroprevalence studies in representative samples of the population must be developed to detect the areas and population groups with insufficient measles protection levels [25].

Supplementary immunization activities (SIAs) can be developed to reach populations and areas with low percentages of vaccination coverage. SIAs based on catch-up and follow-up vaccination campaigns are currently implemented in countries with inadequate first and/or second measles vaccine dose coverage [20]. In 2022, 115 million people received measles vaccines through SIAs in 44 countries [4]. Catch-up SIAs include children aged 9 months−14 years and follow-up SIAs include children aged 9−59 months. SIAs are implemented to provide a second vaccination opportunity to an entire cohort with low measles vaccination coverage, and to protect children who are susceptible to measles due to primary MCV1 failure [2,20]. However, the strategy to increase measles vaccination coverage with SIAs has important economic and logistic limitations that make it insufficient for achieving and maintain measles elimination, such as their inability to detect and vaccinate all susceptible children [10,11,25]. A recent study [41] assessing the quality and results achieved with SIAs implemented since 2020 found a mean SIA coverage among previously measles-zero-dose children was 58.3%, although only 23% of the 66 countries with a national-level SIA had a post-campaign coverage survey report available, and 50% of the reports included the coverage achieved among previously measles-zero-dose children [41]. The analysis of a high-quality anonymized database from an SIA carried out in Somalia in 2022 found that 94.6% of the children included in SIAs had been previously vaccinated with either one or 2+ measles doses, 5.4% of the children had received previously zero doses of measles vaccine, and the SIA coverage was 92% among previously vaccinated children and only 37.2% among zero-dose children [41].

Parental attitudes and beliefs toward measles vaccination are of great importance in influencing measles vaccination [42,43]. Parental hesitancy to vaccinate their children can be attributed to concerns about vaccine efficacy and safety, measles susceptibility, measles severity, vaccine accessibility and mistrust in experts [33,34,35]. Health education activities and national vaccination information campaigns can be developed to fight against measles vaccination hesitancy to reduce misinformation about measles vaccine efficacy and safety and to improve trust in vaccines, health providers and vaccination programs. In the United States of America, National Immunization Awareness Month is an annual observance held in August to highlight the importance of vaccination for people of all ages [36]. These interventions can help raise awareness about the importance of vaccination and encourage people to talk to a healthcare provider they trust about staying up to date on their vaccinations.

Delaying measles elimination in countries and WHO regions makes it more difficult to achieve and maintain measles elimination and eradication in the world [44]. The widening measles vaccination coverage and measles immunity gaps among countries and WHO regions found in this study for the 2019−2023 period shows that the risk of measles outbreaks has increased in all countries and the probability of re-establishing measles transmission in countries where measles had been eliminated is now greater than in 2019.

This study has several limitations. Firstly, the analysis carried out in this study used the information on routine vaccination of the WHO/UNICEF global and regional immunization systems. However, the information reported by the WHO/UNICEF global and regional immunization systems is validated periodically by the World Health Organization [17,18,45]. Secondly, herd immunity levels against measles viruses in different WHO regions were assessed by comparing the mean prevalence of individuals with vaccine-induced measles protection and the critical prevalence blocking the transmission of measles viruses in the population. Measles protection levels would be greater assuming greater levels for measles vaccination effectiveness. However, values of measles vaccination effectiveness of 95% and 92% for two and one doses of measles vaccines, respectively, have been found in studies assessing measles vaccination effectiveness [19]. Thirdly, anti-measles herd immunity levels in different WHO regions were assessed against measles viruses with R_o_ values from 10 to 20. However, this range of R_o_ values has been obtained in studies assessing the transmissibility of measles viruses [10,46].

## 5. Conclusions

The study found that worldwide percentages of two-dose measles vaccination coverage were lower than ≥95% and anti-measles herd immunity levels in the target vaccination population were sufficient to block the transmission of measles viruses with greater transmissibility (Ro ≥ 15) only in the Western Pacific and European WHO regions. All measles vaccination coverage-related and anti-measles herd immunity-related indicators worsened from 2019 to 2023. The zero-dose measles coverage and number of zero-dose measles children found in 2023 were not on track to achieve the AI2030 objective. Interventions to increase routine two-dose measles vaccination coverage should be developed in all WHO regions to meet the goal of eradicating measles worldwide.

## Figures and Tables

**Table 1:** Mean percentages of vaccination coverage for the MCV1 and MCV2 vaccines in 2023 and the MCV1 vaccine in 2021 in the world and WHO regions.

	MCV1 Vaccine 2023	MCV2 Vaccine 2023	MCV1 Vaccine 2021	*n*
	%	%	%	
World	85.2	77.1	83.3	195
African region (AFR)	71.5	62.0	65.0	47
Americas region (AMR)	93.0	83.0	87.5	35
Eastern Mediterranean region (EMR)	91.5	86.5	80.0	22
European region (EUR)	90.5	92.5	93.0	53
South-East Asia region (SEAR)	62.5	54.5	98.0	11
Western Pacific region (WPR)	97.0	95.5	97.5	27

*n*: number of countries with vaccine in national routine vaccination programs; MCV1: first dose of measles-containing vaccine; MCV2: second dose of measles-containing vaccine.

**Table 2:** Mean vaccination coverage with zero, one and two doses of measles vaccines, mean prevalence of individuals in the target vaccination population with vaccine-induced measles protection, percentage of countries with anti-measles herd immunity established in the target population vaccination, and percentage of countries with other measles vaccination indicators in regions of the WHO in 2023.

	World	African Region	Americas Region	Eastern Mediterranean Region	European Region	South-East Asia Region	Western Pacific Region
No. of countries	195	47	35	22	53	11	27
Routine measles vaccination-related indicators							
Mean vaccination coverage (%) with two, one and zero doses of measles vaccine							
2 doses	65.3	48.1	72.5	69.1	86.0	53.0	93.2
1 dose	27.8	30.7	25.5	28.2	13.5	46.5	6.7
0 doses	6.9	21.1	2.0	2.6	0.5	0.5	0.2
Percentage of countries with two-dose measles vaccination coverage ≥ 95% and ≥90%							
≥95%	8.7	0	2.9	22.7	9.4	18.2	14.8
≥90%	21.0	0	8.6	40.9	30.2	36.4	33.3
Percentage of countries where all children had received one or two doses of measles vaccine (0% of zero-dose children)							
	9.7	0	5.7	22.7	9.4	18.2	18.5
Anti-measles herd immunity-related indicators							
Mean prevalence (%) of individuals in the target vaccination population with vaccine-induced measles immunity							
Measles immunity	87.6	74.0	92.3	91.6	94.1	93.1	94.6
Percentage of countries with herd immunity against measles viruses with R_o_ from 10 to ≥20							
R_o_ ≤ 10	66.1	40.4	62.9	68.2	92.4	72.7	59.3
R_o_ ≤ 12	59.0	27.6	54.3	59.1	88.7	63.6	59.3
R_o_ ≤ 15	41.5	10.6	25.7	50.0	66.0	63.6	51.8
R_o_ ≤ 18	23.1	0	8.6	45.4	35.8	36.4	33.3
R_o_ ≤ 19	0	0	5.7	31.8	13.2	27.3	22.2
R_o_ ≥ 20	0	0	0	0	0	0	0

**Table 3:** Variation for measles vaccination and anti-measles herd immunity indicators in different regions of the WHO between 2019 and 2023.

	World	African Region	Americas Region	Eastern Mediterranean Region	European Region	South-East Asia Region	Western Pacific Region
No. of countries	195	47	35	22	53	11	27
Routine measles vaccination-related indicators							
Mean vaccination coverage (%) with two. one and zero doses of measles vaccine							
2 doses	−3.7	24.0	−1.0	−3.2	2.5	−35.0	30.9
1 dose	7.8	−30.7	5.8	27.6	−11.2	171.9	−71.0
0 doses	7.8	24.9	−25.9	−60.0	−44.4	−64.3	−96.5
Percentage of countries with two-dose measles vaccination coverage ≥ 95% and ≥90%							
≥95%	−39.6	−100.0	−79.7	−4.6	−37.7	−33.3	−33.3
≥90%	−18.6	−100.0	−62.4	7.3	−15.6	0.0	−10.0
Percentage of countries where all children had received one or two doses of measles vaccine (0% of zero-dose children)							
	−24.8	−100.0	−60.1	−20.6	0.0	−33.3	0.0
Anti-measles herd immunity-related indicators							
Mean prevalence (%) of individuals in the target vaccination population with vaccine-induced measles immunity							
Measles immunity	−0.6	−4.6	0.5	3.9	0.4	−0.1	6.5
Percentage of countries with herd immunity against measles viruses with R_o_ from 10 to ≥20							
R_o_ ≤ 10	−6.4	0.0	−15.3	2.2	−2.0	−11.1	−15.8
R_o_ ≤ 12	−7.7	−7.4	−17.4	−4.5	−2.1	−12.5	−11.1
R_o_ ≤ 15	−19.4	−28.9	−43.8	−4.6	−18.6	16.7	−17.8
R_o_ ≤ 18	−28.9	−100.0	−62.4	19.2	−13.7	−33.2	−18.2
R_o_ ≤ 19	−100.0	−100.0	−66.7	11.2	−22.4	0.0	0.0
R_o_ ≥ 20	0.0	0.0	0.0	0.0	0.0	0.0	0.0

## Data Availability

The original contributions presented in this study are included in the article. Further inquiries can be directed to the corresponding author.
